# The Fever at Brunswick

**Published:** 1893-10

**Authors:** 


### THE FEVER AT BRUNSWICK.

The situation at Brunswick is well known to our readers. The quarantine was raised and Brunswick was free and happy. The people flocked home and began to repair their losses. Bottled-up Yellow Jack leaped upon its victims and feasted. How hopeful and full of joy was the return to home of the late panic stricken refugee can only be surmised by those who have never experienced an enforced exile. How unwise and how fatal was the return the whole world knows and must remember a long time.

The second outbreak of the epidemic has been a sad blow to Brunswick. At the time of this writing there have been 138 cases of the fever; 82 are now under treatment; 42 have been discharged and 14 have died. The end is not yet. New cases will develop and other deaths will occur until frost comes to the rescue. The lesson of such epidemics is simply this,—that Georgia should hasten to provide herself with an efficient State Board of Health. Brunswick is without the necessary facilities to maintain ordinary vigilance and is wholly unprepared to cope with such an adversary within her gates. The government officers are comparative strangers, but with duties as rigid and inexorable as those of a vidette in time of war. With a hearty and sympathetic co-operation between the people and the officers this chapter of history need never have been written. From the very beginning there was more or less friction which continued and increased until a public sentiment demanded that Brunswick should be made free again and her gates opened to the world. Reluctantly and not without warning the demand was granted.

The opinion of the best informed in the matter and least prejudiced was not heeded and perhaps was not desired. The neighboring islands and the cities in the interior have been invaded by "genuine yellow fever" and no doubt it was carried from Brunswick, though how cannot be explained. Dr. Guiteras, when he left Brunswick, was inclined to the belief that a closed store still held the nucleus of yellow fever and was afraid of its spread whenever the store might be reopened and used. What the limit of this epidemic may be no man can know.

